# Atypical Radiologic Image Characterized by Cavitary Lung Lesions in a Case of Hodgkin Lymphoma

**DOI:** 10.4274/tjh.galenos.2018.2018.0115

**Published:** 2019-02-07

**Authors:** Mahmut Büyükşimşek, Semra Paydaş, Derya Gümürdülü, Cem Mirili, Ali Oğul, Abdullah Evren Yetişir, Mert Tohumcuoğlu

**Affiliations:** 1 Çukurova University Faculty of Medicine, Department of Oncology, Adana, Turkey; 2 Çukurova University Faculty of Medicine, Department of Pathology, Adana, Turkey

**Keywords:** Hodgkin lymphoma, Cavitary lung lesions, Tuberculosis

## To the Editor,

A 30-year-old woman was admitted to the hospital with a lump in her neck. She had no B symptoms (fever, night sweats, and weight loss) and a biopsy showed Hodgkin lymphoma (HL) of the classical type. Positron emission tomography/computed tomography (PET/CT) showed cervical and mediastinal lymph nodes of 1.5-3 cm in diameter and an invasive left parasternal mass of 4x2.5 cm.

Three cycles of ABVD (doxorubicin, bleomycin, vinblastine, dacarbazine) were given and less than partial response (PR) was detected by PET/CT. Salvage chemotherapy and autologous stem cell transplantation (ASCT) were planned and 2 cycles of the DHAP regimen (cisplatin, dexamethasone, cytosine) were given. PR was detected by PET-CT, but she rejected the ASCT. Local radiotherapy was given for the residual tumor. After radiation there was no evidence of a tumor upon PET/CT imaging. One and a half years after the end of radiation she was admitted with cough, dyspnea, sputum, and fever.

**Figure 1:**
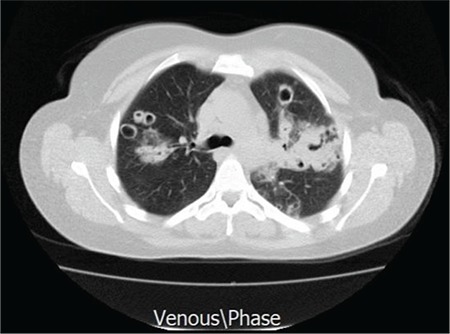
Cavitary pulmonary lesions and atelectasis in the left lingula.

**Figure 2:**
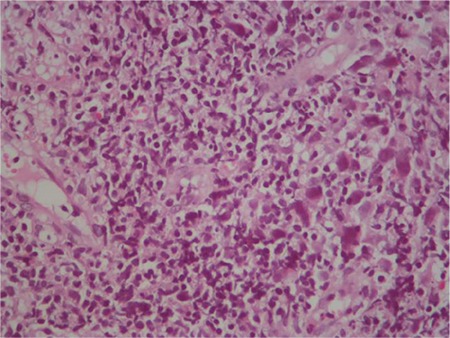
Bronchoscopic biopsy: Hodgkin lymphoma-classical type (H&E, 200^x^).

**Figure 3:**
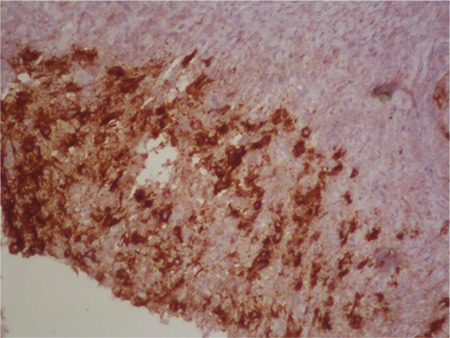
Bronchoscopic biopsy: Hodgkin lymphoma, CD30^+^.

Thoracic CT showed cavitary lesions in the parenchyma of both lungs and atelectasis in the left lingula (Figure 1). The patient was counseled in the department of chest diseases; radiation pneumonia was not considered. The angiotensin-converting enzyme level for sarcoidosis was normal. Bronchoscopic examination, lavage, and biopsy were done. Fungal tests were found to be negative. The cavitary lesion was preferred for biopsy. The biopsy showed HL of the classical type (Figure 2) and CD30 was positive (Figure 3). A QuantiFERON test of the blood sample and tuberculosis polymerase chain reaction from biopsy material were negative.

HL is not a leading diagnostic consideration when evaluating cavitary lung lesions. An extensive differential diagnosis includes vasculitis, infection, and malignancy [1]. Parenchymal lung involvement is not uncommon in HL; however, cavitary pulmonary lesions are quite unusual. Lung involvement in lymphoma is generally seen as nodule formation or consolidation. Bronchoscopic evaluation is very important in these cases [2,3]. Disseminated cavitary lesions mimicking tuberculosis or other opportunistic infections in a case of HL is interesting and differential diagnosis is very important.
